# Supplementary material for: A fault-intrusion-tolerant system and deadline-aware algorithm for scheduling scientific workflow in the cloud
Source: PeerJ Comput Sci. 2021 Nov 2;7:e747. doi: 10.7717/peerj-cs.747 (PMC8576554; doi:10.7717/peerj-cs.747)
Supplement: Supplemental Information 6 [file peerj-cs-07-747-s006.zip › Myproject1.1/docs/org/workflowsim/failure/class-use/FailureRecord.html]

Uses of Class org.workflowsim.failure.FailureRecord


JavaScript is disabled on your browser.


- Overview
- Package
- Class
- Use
- Tree
- Deprecated
- Index
- Help

- Prev
- Next

- Frames
- No Frames

- All Classes

## Uses of Class org.workflowsim.failure.FailureRecord

- Packages that use FailureRecord

  | Package | Description |
  | --- | --- |
  |  |  |
  | --- | --- |
  | org.workflowsim.failure |  |
- - ### Uses of FailureRecord in org.workflowsim.failure

    Fields in org.workflowsim.failure with type parameters of type FailureRecord

    | Modifier and Type | Field and Description |
    | --- | --- |
    |  |  |
    | --- | --- |
    | `protected static java.util.Map<java.lang.Integer,FailureRecord>` | FailureMonitor.`jobid2record` JobID to a Failure Record. |
    | `protected static java.util.List<FailureRecord>` | FailureMonitor.`recordList` All the record list. |
    | `protected static java.util.Map<java.lang.Integer,java.util.ArrayList<FailureRecord>>` | FailureMonitor.`type2record` Type to a Failure Record. |
    | `protected static java.util.Map<java.lang.Integer,java.util.ArrayList<FailureRecord>>` | FailureMonitor.`vm2record` VM ID to a Failure Record. |

    Methods in org.workflowsim.failure with parameters of type FailureRecord

    | Modifier and Type | Method and Description |
    | --- | --- |
    |  |  |
    | --- | --- |
    | `static int` | FailureMonitor.`getClusteringFactor(FailureRecord record)` Gets the clustering factor |
    | `static void` | FailureMonitor.`postFailureRecord(FailureRecord record)` A post from a broker so that we can update record list |

- Overview
- Package
- Class
- Use
- Tree
- Deprecated
- Index
- Help

- Prev
- Next

- Frames
- No Frames

- All Classes
